# Influence of glycemic control on the levels of subgingival periodontal pathogens in patients with generalized chronic periodontitis and type 2 diabetes

**DOI:** 10.1590/1678-77572016-0302

**Published:** 2017

**Authors:** Tamires Szeremeske MIRANDA, Magda FERES, Belén RETAMAL-VALDÉS, Paula Juliana PEREZ-CHAPARRO, Suellen Silva MACIEL, Poliana Mendes DUARTE

**Affiliations:** 1 Universidade Guarulhos, Centro de Pós-Graduação e Pesquisa, Guarulhos, SP, Brasil.

**Keywords:** Chronic periodontitis, Diabetes mellitus, Pathogens, Real-time polymerase chain reaction

## Abstract

**Objective:**

This study evaluated the influence of glycemic control on the levels and frequency of subgingival periodontal pathogens in patients with type 2 diabetes mellitus (DM) and generalized chronic periodontitis (ChP).

**Material and Methods:**

Fifty-six patients with generalized ChP and type 2 DM were assigned according to the levels of glycated hemoglobin (HbA1c) into one of the following groups: HbA1c<8% (n=28) or HbA1c≥8% (n=28). Three subgingival biofilm samples from sites with probing depth (PD)<5 mm and three samples from sites with PD≥5 mm were analyzed by quantitative Polymerase Chain Reaction (PCR) for the presence and levels of *Porphyromonas gingivalis*, *Tannerella forsythia*, *Treponema denticola*, *Eubacterium nodatum*, *Parvimona micra*, *Fusobacterium nucleatum* ssp. and *Prevotella intermedia*.

**Results:**

The mean counts of *F. nucleatum* ssp. were statistically significantly higher in the sites with PD≥5 mm of the HbA1c≥8% group (p<0.05). Frequencies of detection of *T. forsythia*, *E. nodatum*, *P. micra* and *F. nucleatum* ssp. were all higher in the sites with PD≥5 mm of the patients with HbA1c≥8%, compared with those of patients with HbA1c<8% (p<0.05). Frequency of detection of *P. intermedia* was higher in the sites with PD<5 mm of the patients with HbA1c≥8% than those of the patients with HbA1c<8% (p<0.05).

**Conclusions:**

Poor glycemic control, as indicated by HbA1c≥8%, is associated with increased levels and frequencies of periodontal pathogens in the subgingival biofilm of subjects with type 2 DM and ChP.

## Introduction

Diabetes mellitus (DM) has been recognized as a major risk factor for periodontitis, since patients with DM present higher prevalence and severity of periodontal diseases, compared with those without DM. Patients with DM and persistent poor glycemic control have an even higher risk of periodontitis than those with good glycemic control. In addition, effective control of glycemia improves periodontal parameters in patients with type 2 DM and periodontitis^9,14^.

Several mechanisms, including alterations in the host response and in the composition of the subgingival microbiota, have been proposed to explain greater susceptibility of subjects with DM to periodontal breakdown, particularly in patients with poorly controlled DM. Some studies have compared the impact of glycemic control on the immune-inflammatory aspects of sites with periodontitis in patients with type 2 DM. In general, results have shown that periodontal sites of subjects with type 2 DM presenting poor glycemic control are associated with pro-inflammatory and pro-osteoclastogenic profiles^7,21,22^.

Regarding microbiological aspects, previous studies have compared levels of periodontal pathogens in individuals with and without DM using different microbiological techniques^1,6,8,20,23,29,30^. However, to date, few investigations have focused on the impact of glycemic control on the subgingival microorganisms of patients with DM^1,10,17,20,24,28^. Furthermore, most of these studies assessed the occurrence of a limited number of pathogens, especially the fungal kingdom, and focused on the evaluation of subjects with type 1 DM. Therefore, the possible effect of the glycemic control on pathogenic bacterial species in the subgingival biofilm of patients with type 2 DM and periodontitis is not well established. Thus, the aim of this study is to evaluate the impact of glycemic control on frequencies and levels of seven periodontal pathogens (*Treponema denticola, Porphyromonas gingivalis, Tannerella forsythia, Eubacterium nodatum, Parvimona micra, Fusobacterium nucleatum ssp.* and *Prevotella intermedia*) in the subgingival biofilm samples of patients with generalized chronic periodontitis (ChP) and type 2 DM.

## Material and methods

### Subject population

Fifty-six subjects with type 2 DM and generalized ChP^3^ were selected among 390 volunteers screened from the population referred to the Dental Clinic of Guarulhos University, from July 2011 to January 2015. Detailed medical and dental records were obtained. Of the 390 individuals screened, 334 were excluded from participating in the study since they did not meet inclusion criteria. All eligible subjects were informed of the nature, potential risks and benefits of their participation in the study and signed an informed consent form. The Guarulhos University Clinical Research Ethics Committee approved the study protocol.

### Inclusion and exclusion criteria

Inclusion criteria were: ≥35 years of age, diagnosis of type 2 DM for ≥5 years, DM treatment with insulin supplementation, diet regime and/or oral hypoglycemic agents, at least 15 teeth (excluding third molars and teeth with advanced decay indicated for extraction), more than 30% of the sites with probing depth (PD) and clinical attachment level (CAL) ≥4 mm and a minimum of six teeth with at least one site with PD and CAL≥5 mm and bleeding on probing (BoP).

Exclusion criteria were: pregnancy, lactation, current smoking and smoking within the past five years, SRP in the previous 12 months, use of systemic antibiotic, anti-inflammatory and immunosuppressive medications during the previous six months, continuous use of mouthrinses containing antimicrobials in the preceding three months, systemic conditions (except DM) that could affect the progression of periodontitis (e.g., immunological disorders, osteoporosis), need for extensive prosthetic rehabilitation and major complications of DM (i.e., cardiovascular and peripheral vascular diseases [ulcers, gangrene and amputation], neuropathy and nephropathy).

### Experimental groups

Blood samples of all subjects included in the study were examined at the Clinical Analysis Laboratory of Guarulhos University. Fasting plasma glucose (FPG, mg/dL) was measured by the glucose oxidase method and glycated hemoglobin (HbA1c, %) was determined by high-performance liquid chromatography. Fifty-six patients with generalized ChP and type 2 diabetes were assigned according to their levels of HbA1c into one of the following groups: HbA1c<8% (n=28) or HbA1c≥8% (n=28).

### Clinical examination

The study examiner (T.M.S.) participated in a calibration exercise, and standard error of measurement was calculated. Intra-examiner variability was 0.21 mm for PD and 0.24 mm for CAL. Agreement for categorical variables [e.g., BoP] was >85% (Kappa-light test). Visible plaque (presence/absence), BoP (presence/absence), suppuration (presence/absence), PD (mm) and CAL (mm) were assessed at six sites *per* tooth excluding third molars using the manual periodontal probe (North Carolina - Hu-Friedy, Chicago, IL, USA).

### Microbiological analyses

Six subgingival biofilm samples per patient were collected from non-contiguous interproximal sites presenting no furcation involvement; three sites from each of the following PD categories: PD<5 mm and PD≥5 mm. After supragingival plaque removal, biofilm was collected using individual sterile mini-Gracey curettes and placed in individual microtubes containing 0.15 mL of TE (10 mM Tris-HCl, 1 mM EDTA, pH 7.6).

### Quantitative Polymerase Chain Reaction Test (qPCR)

Samples were individually analyzed for the presence and levels of seven bacterial species (*Treponema denticola, Porphyromonas gingivalis, Tanerella forsythia, Eubacterium nodatum, Parvimona micra, Fusobacterium nucleatum ssp.* and *Prevotella intermedia*) by qPCR, using LightCycler 2.0 systems (Roche Diagnostics GmbH, Mannheim, Germany).

**Figure 1:**
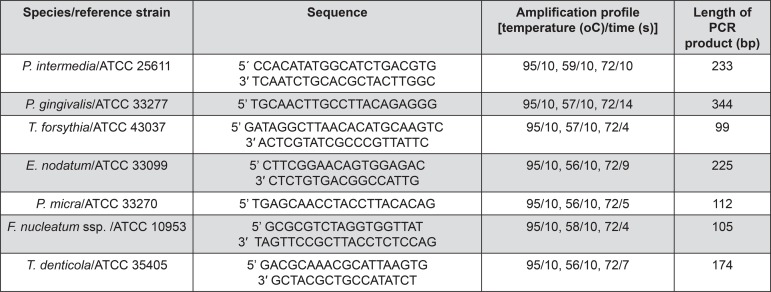
Primer sequences, amplification profile and estimated length of PCR product for each bacterial species

Initially, DNA was extracted from each biofilm sample using the MasterPure^TM^ complete DNA and RNA purification kit (Epicentre, Madison, WI, USA). Amplification reactions were performed in a 10 μL final volume, containing 2.5 μL of the isolated DNA (20 ng/μL) and a reaction mixture containing primer probe sets (2.5 μM each) and FastStart DNA Master SYBR Green I (Roche Diagnostics GmbH, Mannheim, Germany). Absolute quantification of target species in each sample was performed using standard curves prepared with reference strains (Figure 1). The determination of DNA content in controls was based on the genome size of each species and the mean weight of one nucleotide pair^4^. Based on standard curves, individual sample Ct scores were converted into the number of bacterial cells. The level of detection was set to 10^3^ bacteria. Polymerase Chain Reaction (PCR) procedures were performed in a blinded fashion. Specific primers used for the detection of each bacterial species evaluated and the amplification profiles are described in Figure 1.

ATCC: American Type Culture Collection

### Statistical analysis

The minimum number of subjects included in this study was based on a previous study that compared the levels of red complex species between poorly and well-controlled patients with type 2 DM using qPCR^1^. Data were examined for normality by Shapiro-Wilk test and parametric methods were used for data that achieved normal distribution. Clinical, glycemic and microbiological parameters were computed per subject and across subjects in both groups. Significance of differences between groups for age, duration of DM, clinical parameters, bacterial levels and glycemic parameters were compared by Student’s t-test. Fisher’s exact test compared the frequency of gender and DM treatment between groups. The Chi-square test was used to compare the frequency of sites with PD<5 mm and sites with PD≥5 mm colonized by pathogens. Correlations between the levels of HbA1c and the levels of each periodontal pathogen considering all sites and the sites with PD<5 mm and with PD≥5 mm separately were performed using Pearson’s Correlation. Level of significance was set at 5%.

## Results

Table 1 presents demographic characteristics, glycemic and clinical parameters for both groups. There were no statistically significant differences between groups for gender, age, duration of DM and for all clinical parameters (p>0.05). As expected, the HbA1c<8% group presented significant lower levels of HbA1c and FPG than the HbA1c≥8% group (p<0.05). DM treatment reported by the patients of both groups included diet regimen and use of oral hypoglycemic agent (metformin and/or glibenclamide). The frequency of patients that were using the combination of metformin and glibenclamide and the single-drug treatments did not differ between groups (p>0.05).

**Table 1:** Demographic characteristics, glycemic parameters (mean ± SD) and full-mouth clinical parameters (mean ± SD) of both groups of the study population

Parameters	HbA1c< 8%	HbA1c≥ 8%	p-value
Gender (M/F; n)	18/16	12/16	0.11
Age (mean ± SD; years)	55.4±6.1	51.7±8.7	0.07
Duration of DM (years)	5.0±5.8	5.0±6.7	0.98
Hb1A (%)	6.9±1.0	9.9±1.5	0.02
FPG (mg/dl)	122±31	177±32	0.04
Sites with plaque accumulation (%)	81.0±16.4	73.1±25.3	0.17
Sites with bleeding on probing (%)	37.1±13.2	37.5±16.8	0.93
Mean PD (mm)	3.8±0.7	3.5±0.5	0.14
Mean CAL (mm)	4.7±1.3	4.5±0.8	0.56
Sites with PD ≥5mm (%)	27.3 ±13.7	27.1 ±11.7	0.83

PD: probing depth; CAL: clinical attachment level; HbA1c: glycated hemoglobin; FPG: fasting plasma glucose; SD: standard deviation; A p-value <0.05 indicates differences between groups by the Student’s t-test (p<0.05). There were no differences between groups for gender by the Fisher's exact test (p>0.05).

Table 2 presents counts of periodontal pathogens for all sites and for sites with PD<5 mm and with PD≥5 mm for both groups. There were no differences in mean numbers of almost all bacterial species between groups, considering all sites as well as sites with PD<5 mm and PD≥5 mm (p>0.05). Mean levels of *F. nucleatum ssp.* were statistically significantly higher in the sites with PD≥5 mm of the HbA1c≥8% group than in those of the HbA1c<8% group (p<0.05).

**Table 2:** Mean (±SD) counts (log10) of periodontal pathogens for all sites and for sites with PD < 5 mm and ≥5 mm for both groups

Species	Sites	HbA1c<8%	HbA1c≥8%	p-value
*T. denticola*	All PD	4.7±1.3	4.4±1.4	0.17
	PD <5 mm	4.6±1.4	4.0±1.6	0.19
	PD ≥5 mm	5.1±1.5	5.1±1.5	0.34
*P. gingivalis*	All PD	3.7±1.4	3.8±1.6	0.44
	PD <5 mm	3.4±1.6	3.4±1.8	0.88
	PD ≥5 mm	4.5±2.2	4.6±1.8	0.79
*T. forsythia*	All PD	3.9±1.2	4.0±1.0	0.22
	PD <5 mm	3.8±1.3	3.5±1.2	0.45
	PD ≥5 mm	4.3±1.6	4.8±0.9	0.12
*E. nodatum*	All PD	3.8±1.1	3.7±0.9	0.35
	PD <5 mm	3.7±1.1	3.3±1.1	0.71
	PD ≥5 mm	3.9±1.1	4.4±0.8	0.11
*P. micra*	All PD	4.2±1.2	4.3±1.2	0.23
	PD <5 mm	4.2±1.2	3.9±1.4	0.42
	PD ≥5 mm	4.3±1.7	4.9±1.7	0.13
*F. nucleatum ssp.*	All PD	4.7±1.2	4.8±1.0	0.29
	PD <5 mm	4.7±1.3	4.5±1.3	0.58
	PD ≥5 mm	4.6±1.5	5.4±0.7	0.02
*P. intermedia*	All PD	3.8±1.4	3.9±1.5	0.83
	PD <5 mm	3.7±1.5	3.8±1.7	0.25
	PD ≥5 mm	4.0±1.5	4.0±1.5	0.91

SD: standard deviation; A p-value <0.05 indicates differences between groups; Student’s t-test (p<0.05).

The percentages sites with PD<5 mm and PD≥5 mm colonized by periodontal pathogens for both groups are presented in Table 3. Frequencies of detection of *T. forsythia, E. nodatum, P. micra* and *F. nucleatum ssp* were higher in the sites with PD≥5 mm of the HbA1c≥8% group, compared with the HbA1c<8% group (p<0.05). Frequency of detection of *P. intermedia* was higher in sites with PD<5 mm of the HbA1c≥8% group, compared with the HbA1c<8% group (p<0.05).

**Table 3:** Percentage of sites with PD <5 mm and ≥5 mm colonized by periodontal pathogens for both groups

		Groups		
Species	Sites	HbA1c < 8%	HbA1c ≥ 8%	p-value
*T. denticola*	PD <5 mm	90.00%	82.90%	0.11
	PD ≥5 mm	87.50%	96.40%	0.08
*P. gingivalis*	PD <5 mm	79.30%	76.60%	0.63
	PD ≥5 mm	80.30%	91.00%	0.1
*T. forsythia*	PD <5 mm	88.30%	87.40%	0.83
	PD ≥5 mm	83.90%	96.40%	0.02
*E. nodatum*	PD <5 mm	89.20%	85.60%	0.42
	PD ≥5 mm	82.10%	96.40%	0.01
*P. micra*	PD <5 mm	91.90%	89.20%	0.15
	PD ≥5 mm	83.90%	98.20%	0.008
*F. nucleatum* ssp.	PD <5 mm	90.90%	90.00%	0.81
	PD ≥5 mm	83.90%	98.20%	0.008
*P. intermedia*	PD <5 mm	73.00%	90.00%	0.007
	PD ≥5 mm	83.90%	92.80%	0.14

A p-value <0.05 indicates differences between groups (Chi-square test)

According to Table 4, in the HbA1c≥8% group, counts of *P. micra* and *T. forsythia* in the sites with PD<5 mm presented weak positive correlations with levels of HbA1c (p<0.05). Furthermore, counts of *P. intermedia* in the sites with PD≥5 mm of the HbA1c≥8% group exhibited moderate positive correlation with levels of HbA1c (p<0.05).

**Table 4:** Correlation coefficients (r) for the relationship between each periodontal pathogen and HbA1c levels considering all sites and sites with PD <5 mm and ≥5 mm

	HbA1c < 8% group													
	*T. denticola*		*P. gingivalis*		*T. forsythia*		*E. nodatum*		*P. micra*		*F. nucleatum* ssp		*P. intermedia*	
	r	p	r	p	r	p	r	p	r	p	r	p	r	p
All sites HbA1c	0.051	0.796	0.087	0.658	0.153	0.435	0.13	0.508	0.135	0.491	0.046	0.814	-0.044	0.822
Sites with PD <5 mm HbA1c	-0.047	0.812	-0.015	0.936	0.092	0.639	0.091	0.642	0.052	0.789	0.027	0.888	-0.082	0.676
Sites with PD ≥5 mm HbA1c	0.219	0.261	0.213	0.274	0.202	0.3	0.169	0.388	0.229	0.239	0.057	0.77	0.016	0.932

	**HbA1c ≥ 8% group**													
	***T. denticola***		***P. gingivalis***		***T. forsythia***		***E. nodatum***		***P. micra***		***F .nucleatum* ssp**		***P. intermedia***	
	**r**	**p**	**r**	**p**	**r**	**p**	**r**	**p**	**r**	**p**	**r**	**p**	**r**	**p**

All sites HbA1c	0.023	0.906	0.269	0.165	0.338	0.078	0.3	0.12	0.368	0.053	0.29	0.133	0.192	0.325
Sites with PD <5 mm HbA1c	0.111	0.573	0.341	0.074	0.369	0.042	0.333	0.082	0.376	0.048	0.286	0.139	0.246	0.206
Sites with PD ≥5 mm HbA1c	-0.166	0.396	0.067	0.735	0.156	0.427	0.062	0.75	0.276	0.155	0.23	0.237	0.583	0.044

Pearson Correlation (p<0.05)

## Discussion

This study evaluated the frequency of detection and levels of seven periodontal pathogens in the subgingival biofilm of poorly and better-controlled patients with type 2 DM and ChP. Results indicated that poorly-controlled subjects, defined as HbA1c≥8%^11,18^, harbored higher counts of *F. nucleatum* in the sites with PD≥5 mm, compared with better controlled patients (HbA1c<8%). Moreover, periodontal sites of individuals with critical glycemic control demonstrated an increased frequency of detection of *T. forsythia* and of the four orange complex species studied, when compared with those of patients with better-controlled glycemia. These findings suggest that poor glycemic control in subjects with type 2 DM is associated with a more pathogenic subgingival microbial profile, which could contribute, at least in part, to the worsened periodontitis observed in these subjects^16^. Noteworthy, previous studies from the medical field have shown that patients with type 2 DM presenting inadequate glycemic control can be at higher risk for developing several types of infections^11,13^, corroborating the present evidence demonstrating the possible impact of hyperglycemia on infectious diseases.

Sites with PD≥5 mm of patients with type 2 DM with HbA1c≥8% had higher mean levels and prevalence of *F. nucleatum,* compared with those of the patients with HbA1c<8%. *F. nucleatum* is an anaerobic periodontal pathogen whose prevalence increases as the severity and progression of periodontal diseases increase^27^. The pathogenic activities of *F. nucleatum* involve the production of virulence factors that enable this species to aggregate with other species in mixed infections, to adhere to host molecules and to invade host cells. Because of its ability to aggregate with other suspected pathogens in periodontal diseases, *F. nucleatum* acts as a bridge between early and late colonizers during biofilm formation^12,26^. In support of the current results, Sakalauskiene, et al.^20^ (2014) observed a relationship between the presence of *F. nucleatum* and HbA1c levels in patients with type 1 DM. Therefore, it is supposed that the colonization of periodontal pockets by *F. nucleatum* may be affected by environmental factors such as hyperglycemia. Further studies are required to confirm this hypothesis.

Although *F. nucleatum* was the only species that differed in counts between groups, the prevalence of detection of *T. forsythia, E. nodatum* and *P. micra* were higher in the sites with PD≥5 mm of the patients with HbA1c≥8%. In addition, counts of *P. intermedia* in this category of PD exhibited moderate positive correlation with the levels of HbA1c in poorly controlled subjects. Tervonen, et al.^28^(1994) showed that the duration, type and metabolic control of the subjects with DM (type 1 and type 2) had no significant effect on the prevalence of *Aggregatibacter actinomycetemcomitans, F. nucleatum, Eikenella corrodens, P. gingivalis* and *P. intermedia*. On the other hand, in another previous study, levels of *P. gingivalis, T. denticola* and *T. forsythia* were positively correlated with HbA1c in subjects with type 2 DM^1^. Moreover, the frequency of detection of *T. forsythia* and *T. denticola* was correlated with poorer metabolic control in subjects with type 1 DM^24^. In addition, elevated levels of HbA1c were related to a higher frequency of detection of *P. gingivalis* after the periodontal treatment of subjects with type 2 DM^17^. Divergences among studies regarding the species of periodontal pathogen affected by poor glycemic control may be attributed to differences in type of DM, PD of the sampled sites, severity of hyperglycemia and methods used for bacterial detection. Nevertheless, the majority of this above-mentioned evidence indicates that inadequate glycemic control might influence, to some extent, the colonization of the periodontal pockets of subjects with DM by pathogens. These microbiological results might support previous immunological findings, demonstrating that poor glycemic control is associated with elevated levels of pro-inflammatory cytokines, such as interleukin (IL)-ß, IL-17 and receptor activator of nuclear factor-kappa B (RANKL), and decreased levels of anti-inflammatory cytokines, such as IL-4^7,21,22^.

An important finding of the current study is that sites with PD<5 mm, i.e., the shallower sites, of the subjects with HbA1c≥8% presented a higher frequency of detection of *P. intermedia* than those of subjects with HbA1c<8%. In addition, numbers of *T. forsythia* and *P. micra* in these sites of the poorly-controlled subjects were slightly positively correlated with the levels of HbA1c. *T. forsythia* and *P. intermedia* are classical periodontal pathogens with an arsenal of virulence factors (e.g., proteolytic enzymes, surface-layer and lipoproteins, leucine-rich repeat BspA protein etc.) that stimulates the host immune and inflammatory responses and, consequently, the periodontal breakdown^19,25,27^. Furthermore, *P. micra* has been recognized as a putative periodontal pathogen found in high frequency and levels in ChP lesions that is able to predict the worsening of periodontal parameters^2,27^. Therefore, it seems that the negative influence of glycemic control on the subgingival biofilm is not only limited to sites with PD≥5 mm, but also extends to those with lower PD. Interestingly, a previous study from our group showed that uncontrolled subjects with type 2 DM exhibited a hyper-immune-inflammatory response even in shallow sites^5^. The direct implication of these findings is that shallow sites of patients with unsatisfactory glycemia may be at risk of future periodontal breakdown, due to the bacterial challenge and the exacerbated host response to pathogens. Therefore, it is supposed that sites with PD<5 mm in poorly-controlled patients should receive optimum periodontal therapy similar to that provided for deeper sites.

The main strength of this study was assessment of the prevalence and levels of seven periodontal pathogens, including the three species of the red complex and four species of the orange complex, using a quantitative sensitive method of PCR. In addition, the patients from both groups were matched for age, gender, duration and treatment regimen of DM, as well as for severity of periodontitis at full-mouth and sampled sites levels. Furthermore, HbA1c analysis was performed in the same laboratory using the same method.

Certain limitations should be considered when interpreting the current findings. First, from the current study design, it is not possible to determine the actual mechanisms by which increased level of glycemia/HbA1c might favor increased counts of some pathogens in different PD categories. It is unknown whether hyperglycemia might directly alter the nutritional and environmental factors in the pocket, fostering the colonization/persistence of pathogens that trigger an exacerbated immune-inflammatory response, whether the poor glycemic control stimulates hyper-inflammation in the pocket environment, favoring indirectly the colonization/persistence of pathogens, or both. Therefore, the cyclic or synergic interactions between microbiological and immunological components in the periodontal sites of poorly-controlled patients that increase periodontal destruction should be better elucidated. In addition, although evidence has suggested that periodontal infection may impair adequate glycemic control^15^, the current study is not able to establish whether the observed altered pathogen profile might contribute to increased insulin resistance and poor glycemic control or vice versa. Third, HbA1c was checked only once in each patient, reflecting the glycemic control restricted to the previous one to three months. Furthermore, since this study is limited to only one point in time, the actual clinical consequences of these microbiological findings should be assessed over a longer follow-up of these poorly-controlled subjects. Finally, further evaluations assessing the effects of the glycemic control on the subgingival biofilm of these subjects would be important to add to the current findings.

In conclusion, poor glycemic control, as indicated by HbA1c≥8%, is associated with increased levels and frequencies of periodontal pathogens in the subgingival biofilm of subjects with type 2 DM and ChP.
